# Resting-state EEG activity in depression: a systematic review and *meta*-analysis

**DOI:** 10.1016/j.nicl.2026.103997

**Published:** 2026-04-26

**Authors:** Henrik Heitmann, Jean-François Siani, Paul Theo Zebhauser, Peter Henningsen, Stefan Leucht, Josef Priller, Markus Ploner

**Affiliations:** a Department of Psychosomatic Medicine and Psychotherapy, TUM School of Medicine and Health, Technical University of Munich (TUM), Munich, Germany; b Center for Interdisciplinary Pain Medicine, TUM School of Medicine and Health, Technical University of Munich (TUM), Munich, Germany; c Department of Neurology, TUM School of Medicine and Health, Technical University of Munich (TUM), Munich, Germany; d TUM-Neuroimaging Center, TUM School of Medicine and Health, Technical University of Munich (TUM), Munich, Germany; e Department of Psychiatry, TUM School of Medicine and Health, Technical University of Munich (TUM), Munich, Germany

**Keywords:** EEG, Resting-state, Beta power, Systematic review, Meta-analysis, Biomarker

## Abstract

Depression is a highly prevalent and disabling disorder affecting approximately 5% of the adult population worldwide. Despite its impact, the underlying pathophysiology remains insufficiently understood, and current treatments are only partially effective. Understanding electrophysiological correlates of depression offers promise for a better grasp of the underlying brain mechanisms and might even guide novel treatment approaches, including neuromodulation. EEG is particularly attractive for this purpose due to its wide availability, cost-effectiveness, and potential for direct neuromodulatory targeting.

We conducted a PROSPERO-registered systematic review in accordance with PRISMA guidelines to assess resting-state EEG activity in adult patients with depression, diagnosed according to DSM-IV/V or ICD-10/11. Included studies reported cross-sectional or correlational data on well-established quantitative EEG measures such as power, cordance, peak frequency, and alpha asymmetry. Semiquantitative analyses using modified albatross plots and *meta*-analyses were performed. Study quality was assessed with a modified Newcastle-Ottawa Scale.

Fifty-two studies met the inclusion criteria. Semiquantitative findings showed a trend for increased low-frequency (delta and theta) and high-frequency (beta and gamma) power, as well as left frontal alpha asymmetry, in depressed patients compared to healthy controls. However, *meta*-analysis only confirmed a significant increase in beta power. Results regarding disease severity correlations and data on peak alpha frequency and cordance were insufficient for interpretation. Risk of bias across studies was high.

Our results support a potential role for increased beta oscillations in depression. These oscillations may reflect disrupted corticolimbic control and reward processing and partially overlap with mechanisms implicated in chronic pain and fatigue. Further investigation is warranted into their potential as a diagnostic tool or even a biomarker, as well as their potential use as a neuromodulatory treatment target.

## Highlights

• PROSPERO-registered systematic review in accordance with PRISMA guidelines assessing resting-state EEG activity in depression.
• Meta-analytic evidence for increased beta power in patients with depression.
• Potential as a biomarker and neuromodulation treatment target should be further evaluated.

## 1 Introduction

Depression is a highly prevalent and disabling disorder affecting around 5% of the adult population worldwide, imposing a significant burden on societies and healthcare systems (Collaborators, 2022, Shorey et al., 2022). Despite considerable research, its pathophysiology is not fully clear, and treatments are often insufficient (Nemeroff 2020).

The diagnosis of depression is established according to disease classifications, with the Diagnostic and Statistical Manual of Mental Disorders (DSM) and the International Statistical Classification of Diseases and Health Related Problems (ICD) being the most established. Diagnosis and monitoring of depression according to these frameworks rely on questionnaires and interviews. In addition to these rather subjective assessments, more objective biomarkers for the diagnosis and monitoring of depression appear appealing. According to the National Institutes of Health (NIH) Biomarkers, EndpointS, and other Tools (BEST) classification, biomarkers can fulfill different functions. Diagnostic biomarkers might allow the differentiation of patients with depression from healthy participants, and monitoring biomarkers may enable evaluation and tracking of depression severity, including treatment responses, which might be particularly valuable (FDA-NIH Biomarker Working Group 2016). Since depression is associated with aberrant brain activity (Muller et al., 2017), biomarkers based on brain activity might help to understand the pathophysiology and to develop targeted treatment approaches, e.g., using neuromodulation techniques (de Aguiar Neto and Rosa, 2019, Newson and Thiagarajan, 2019, Marwaha et al., 2023). Using resting-state electroencephalography (EEG) to detect such biomarkers could be of interest, as it is widely available, cost-effective, and potentially scalable (de Aguiar Neto and Rosa 2019). Correspondingly, an increasing number of studies have investigated the potential of various EEG parameters, e.g., as diagnostic biomarkers and predictors of treatment response in depression (Olbrich et al., 2015). Moreover, recent neuromodulatory treatment approaches, such as non-invasive brain stimulation, allow for direct targeting of brain activity captured by EEG, thereby further underlining the translational potential of EEG findings (de Aguiar Neto and Rosa 2019).

Previous systematic reviews point towards altered EEG power, asymmetry, and connectivity in patients with depression, with an increase in low-frequency band power and alpha asymmetry with relatively increased left-sided activity in patients compared to healthy participants being most consistently described (van der Vinne et al., 2017, de Aguiar Neto and Rosa, 2019, Newson and Thiagarajan, 2019, Miljevic et al., 2023, Luo et al., 2025). While these studies provide valuable insights, the wide variety of EEG parameters investigated often hindered quantitative analysis and interpretation of results (e.g., *meta*-analysis) (Miljevic et al., 2023, Tsai et al., 2023). This is particularly true for connectivity measures, thereby fostering the discussion on how the plethora of these measures might compromise interpretability (Jensen et al., 2025). Furthermore, also the heterogeneity of study populations included, often lacking clear diagnostic criteria for depression, makes it challenging to derive clinically meaningful conclusions and approaches.

To address these challenges, the present study aims to provide an overview of basic EEG activity-based measures that could potentially serve as biomarkers or even possible future treatment targets in depression. To this end, the relationship between depression, as defined by DSM-IV/DSM-5 and ICD-10/ICD-11, and longstanding, well-established quantitative activity-related EEG parameters, including band-specific power, alpha asymmetry, peak alpha frequency, and cordance, is investigated. Previous narrative reviews reported an increase in delta, theta, and beta oscillations (Newson and Thiagarajan, 2019). In particular, theta oscillations have been discussed as potential monitoring biomarkers in depression, as they have been found to predict therapeutic response to non-invasive brain stimulation (Bailey et al., 2018), making them a potential treatment target (Tsai et al., 2023). Beta oscillations have been implicated in the top-down control of cortico-limbic circuits in depression (Hoy et al., 2023, Amemori et al., 2024, Xiao et al., 2024) and have been discussed as a potential biomarker of anhedonia (Xiao et al., 2024). Furthermore, oscillations in both frequency bands have been related to reward learning in depression. Alpha asymmetry refers to an increase in left-hemisphere compared to right-hemisphere frontal alpha band power, frequently reported in depression (van der Vinne et al., 2017). Increased alpha activity is associated with inhibitory processes, suggesting a cortical region is less active. Since the activation of the left hemisphere has been suggested to be associated with approach behaviors, while the right hemisphere is associated with withdrawal behaviors, the left hemisphere alpha increase in depression may reflect a bias towards avoidance behavior (van der Vinne et al., 2017). The peak alpha frequency (PAF) refers to the highest frequency in the power spectrum within the alpha frequency band and has been linked to individual responses to pharmacological and brain stimulation treatments in depression (Voetterl et al., 2023). Cordance is a measure of regional brain activity that combines absolute and relative EEG power (Leuchter, 1994, de la Salle et al., 2020). It has been suggested to be particularly closely related to brain metabolism and implicated in treatment response prediction to antidepressant medications and interventions, including brain stimulation (de la Salle et al., 2020). Focusing on such frequently analysed EEG parameters enables a more standardized and reproducible approach with high translational applicability. This holds the potential to contribute to a better pathophysiological understanding of depression, aiding its diagnosis and treatment.

## 2 Methods

The present study was conducted and is reported following the Preferred Reporting Items for Systematic Reviews and Meta-Analyses (PRISMA) (Page et al., 2021). The study protocol was preregistered on PROSPERO (Identifier CRD 42024492853). The process of deduplication, screening of title and abstract, as well as full-text review and data extraction, were performed using the software Covidence (Covidence 2021). The present follows the methodology of previous systematic reviews performed for chronic pain, fatigue and migraine (Heitmann et al., 2023, Zebhauser et al., 2023, Zebhauser et al., 2024).

### 2.1 Search strategy

The databases MEDLINE, PubMed Central, and Bookshelf (through PubMed), Web of Science Core Collection (through Web of Science), and EMBASE (through Ovid) were searched. The search strings used comprised combinations of depression and EEG, using Boolean operators and truncations, and can be found in detail for each database in the supplementary material. Databases were searched from their inception dates until 22 February and again on 5 September 2024. No language restrictions were applied. Additionally, reference mining of recent reviews on EEG in depression (de Aguiar Neto and Rosa, 2019, Newson and Thiagarajan, 2019, Miljevic et al., 2023) and included studies were performed.

### 2.2 Study selection

For detailed inclusion and exclusion criteria, please see Table 1. In summary, cross-sectional and correlational data from peer-reviewed studies measuring quantitative EEG activity measures (power, asymmetry, peak frequency, and cordance) in awake resting-state adult human patients currently suffering from depression according to DSM IV/V (Major Depressive Disorder) or ICD 10/11 (F32. or F33./6A70. or 6A71.) as assessed by a corresponding diagnostic interview were included (for information on diagnostic procedures and severity assessments applied please see Table 2). Studies involving people with other severe neuropsychiatric disorders, including other types of depressive disorders, were excluded.

**Table 1:** Inclusion and exclusion criteria.

Inclusion (if all apply)	Exclusion (if any applies)
Depression (according to DSM IV/V or ICD 10/11) as the primary condition studied	Other severe neuropsychiatric comorbidities incl. substance-induced mood-disorders
Wake resting-state EEG recording in adult humans	
At least one of the following quantitative EEG measures reported - Band-specific power - Band-specific asymmetry - Peak-frequency - Cordance	
Cross-sectional comparison with healthy control group or correlation with disease severity	
Published and peer-reviewed studies	Review articles and case studies

**Table 2:** Detailed study information.

Study ID	Analysis approach (GC/ CORR)	Sample Size MDD (n = )	Age MDD ± SD	Sex (female in %)	Disease duration (years) ± SD	Sample Size Controls (n = )	Age Control ± SD	Sex (female in %)	Diagnostic manual	Severity assmt.	Structured interview/ cutoff used	No. of elec.	Dur. of rec. (min)	Eyes (open/ closed)	Power (global/ local)	Power (absolute/ relative)	Referencing	Delta range (Hz)	Theta range (Hz)	Alpha range (Hz)	Beta range (Hz)	Gamma range (Hz)
Allen 2010	GC	62	n/a	n/a	n/a	163	n/a	65,6	DSM-IV	HDRS	DSM interview	64	8	both	local	absolute	average reference	n/a	n/a	8–13	n/a	n/a
Allen 2004	CORR	29	n/a	100	n/a		n/a	n/a	DSM-IV	HDRS	DSM interview	25	8	both	local	absolute	average reference	n/a	n/a	8–13	n/a	n/a
Arikan 2019	GC	218	38,42 ± n/a	49	n/a	30	43,9 ± n/a	50	DSM-IV&V	BDI	DSM interview	19	7	closed	local	absolute	A1-A2	1–4	4–7	8–12	12–25	30–50
Arns 2016	both	1008	37,84 ± n/a	57	n/a	336	36,99 ± n/a	57	DSM-IV	HDRS	both, DSM + >/=16 on HDRS-17	26	4	both	local	absolute	averaged mastoids	n/a	6.5–8	n/a	n/a	n/a
Arns 2015	both	1008	37,84 ± n/a	57	n/a	336	36,99 ± n/a	57	DSM-IV	HDRS	both, DSM + >/=16 on HDRS-17	26	4	both	global	absolute	averaged mastoids	n/a	n/a	8–13	n/a	n/a
Arns 2015	both	1008	37,84 ± n/a	57	n/a	336	36,99 ± n/a	57	DSM-IV	HDRS	both, DSM + >/=16 on HDRS-17	26	4	closed	local	absolute	averaged mastoids	n/a	n/a	8–13	n/a	n/a
Begić 2011	GC	33	55,1 ± 12,6	n/a	5.2 ± 3.3	30	35,9 ± 10,1	n/a	DSM-IV	n/a	DSM interview	12	1,66	closed	local	absolute	linked ears	0.5–4	4–8	8–13	13–30	n/a
Cantisani 2015	both	20	43,3 ± 14,03	50	n/a	19	41,05 ± 13,82	57,9	DSM-IV	n/a	both, DSM + >/=18 on HDRS-17	70	6	both	global	absolute	average reference	n/a	n/a	8–12.5	n/a	n/a
Cook 2014	both	121	n/a	n/a	n/a	47	n/a	n/a	DSM-IV	HDRS	both, DSM + >/=16 on HDRS-17	35	10	closed	local	relative	A1-A2	n/a	4–8	n/a	n/a	n/a
Čukić 2018	GC	11	44,64 ± 10,87	55	n/a	20	30,14 ± 8,94	50	ICD-10	MDI	ICD interview	19	3	closed	local	absolute	n/a	n/a	n/a	8–12	13–30	n/a
Das 2020	both	30	31,77 ± 10,1	30	n/a	30	32,07 ± 8,76	30	ICD-10	HDRS	ICD interview	25	15	closed	local	absolute	average reference	n/a	4–7.5	7.5–14	14–20	n/a
Dharmadhikari 2019	GC	24	34,82 ± 11,05	63	n/a	17	29,52 ± 9,8	64,7	DSM-V	HDRS	DSM interview	32	10	closed	local	absolute	auricular	n/a	n/a	8–13	n/a	n/a
Escolano 2014	CORR	60	52,3 ± 10,62	68	n/a		n/a	n/a	DSM-V	BDI	DSM interview	16	6	both	global	absolute	average reference	1–4	4.5–7	8–12	12–30	n/a
Fahrabod 2010	CORR	7	52,74 ± 11,64	57	n/a		n/a	n/a	DSM-IV	HDRS	DSM interview	36	0,5	closed	local	absolute	n/a	1–3	3–7	7–11	11–29	n/a
Gold 2013	CORR	79	35,6 ± 9,8	78	n/a		n/a	n/a	ICD-10	MADS	ICD interview	32	5	closed	local	absolute	mastoid, except Cz for FAA	n/a	4–8	8–12	n/a	n/a
Hill 2021	GC	21	46,5 ± 11,5	52	n/a	22	36,14 ± 11,43	31,8	DSM-IV	HDRS	DSM interview	64	10	closed	global	relative	average reference	1–3	4–7	8–12	13–29	30–55
Huang 2023	both	36	22,7 ± 2,1	61	0.5 ± 0.123	31	25,2 ± 6,9	54	DSM-V	HDRS	both, DSM + >/=17 on HDRS-17	32	10	closed	local	relative	average reference	1–4	4–8	8–13	13–30	30–80
Jang 2023	GC	31	37,52 ± 10,79	68	n/a	31	38,97 ± 9,63	48,4	DSM-V	HDRS	DSM interview	62	5	closed	local	absolute	average reference	1–4	4–8	8–12	12–30	30–50
Jaworska 2012	both	53	40,7 ± 11,8	55	n/a	43	36,56 ± 9,97	53,5	DSM-IV	POMS	DSM interview	32	6	both	local	absolute	mastoid, Cz and average	n/a	4–8	8–13	n/a	n/a
Jiang 2023	GC	129	25,62 ± 8,13	66	n/a	60	25,33 ± 4,84	55	DSM-IV	HDRS	both, DSM + >/=20 on HDRS-17	68	5	closed	local	relative	FCz	1–4	4–7	8–12	12–30	30–100
Kemp 2010	both	15	39,9 ± 14	60	n/a	15	42,4 ± 16,7	60	DSM-IV	HDRS	both, DSM + >/=18 on HDRS-17	26	2	closed	local	relative	averaged A1, A2	1.5–3.5	4–7.5	8–13	14.5–30	n/a
Kesebir 2022	CORR	363	29,4 ± 5,1	57	n/a		n/a	n/a	DSM-V	SCL	DSM interview	18	3	closed	local	relative	averaged A1, A2	n/a	n/a	n/a	n/a	n/a
Kim 2019	CORR	38	26,7 ± 8,3	42	n/a		n/a	n/a	DSM-IV	HDRS	DSM interview	62	5	closed	local	relative	Cz, CPz	1–4	4–8	8–12	12–30	30–50
Knott 2000	CORR	69	37,8 ± 10,3	0	n/a		n/a	n/a	DSM-IV	HDRS	both, DSM + >/=18 on HDRS-17	21	10	closed	global	relative	earlobe electrode	1.5–3.5	3.5–7.5	7.5–12.5	12.5–25	n/a
Knott 2001	GC	23	37,8 ± 10,3	0	n/a	23	36,1 ± 9,1	0	DSM-IV	HDRS	both, DSM + >/=18 on HDRS-17	21	20	closed	global	relative	earlobe electrode	1.5–3.5	5–7.5	7.5–12.5	12.5–25	n/a
Koo 2019	GC	20	51,05 ± 10,5	55	0.57 ± 0.15	20	47,15 ± 12,57	65	DSM-IV	HDRS	DSM interview	31	10	closed	local	absolute	average reference	n/a	n/a	8–12	n/a	n/a
Korb 2008	both	37	11,9 ± 38,8	54	n/a	37	37,5 ± 13,1	54,1	DSM-IV	HDRS	both, DSM + >/=16 on HDRS-17	36	20	closed	global	absolute and relative	FpZ & linked Ears	1–3	4–7	8–12	13–30	n/a
Lin 2023	GC	45	21,1 ± 2,6	67	6.10 ± 6.67	46	21,6 ± 2	54,3	DSM-V	BDI	both, DSM + >/=14 on BDI-II + >/=8 BAI	64	8	both	global and local	absolute	average reference	1–4	4–8	8–12	12–32	n/a
Lin 2021	both	137	42,12 ± 14,11	68	6.10 ± 6.67	141	40,32 ± 14,42	66,7	DSM-V	BDI	both, DSM + >/=14 on BDI-II + >/=8 BAI	19	5	closed	local	relative	linked ears	1–4	4–8	8–12	12–32	n/a
Liu 2024	GC	86	26,16 ± 6,29	64	n/a	83	26,41 ± 8,24	55,4	DSM-V	HDRS	both, DSM + >/=17 on HDRS-17	19	20	open	local	relative	average reference	1–4	4–8	8–12	12–40	n/a
Liu 2022a	GC	43	29,7 ± 7,62	74	n/a	57	27,07 ± 7,17	61,4	DSM-V	HDRS	DSM interview	64	8	both	global	relative	average reference	1–4	4–8	8–13	13–30	30–150
Liu 2022b	GC	30	27,13 ± 5,58	73	n/a	26	25,54 ± 1,58	50	DSM-V	HDRS	both, DSM + >/=14 on HDRS-17	64	8	both	global, local for theta band only	absolute	mastoid	0.5–4	4–8	8–13	13–30	30–120
Marcu 2023	CORR	24	45,33 ± 14,63	54	n/a		n/a	n/a	DSM-V	MADS	DSM interview	34	10	closed	local	absolute	Surface Laplacian	n/a	4–8	8–13	n/a	n/a
Morgan 2005	both	76	45,6 ± 16	66	n/a	28	56 ± 18,2	64,3	DSM-IV	HDRS	DSM interview	35	0,5	closed	global	absolute	average reference	0.5–4	4–8	8–12	12–20	n/a
Mumtaz 2016	GC	33	40,33 ± 12,86	n/a	n/a	30	38,23 ± 15,64	n/a	DSM-V	n/a	DSM interview	19	10	closed	local	absolute	infinity reference	n/a	n/a	n/a	n/a	n/a
Pizzagalli 2002	GC	20	34,74 ± 11,24	61	n/a	18	38,6 ± 13,6	55,5	DSM-IV	HDRS	DSM interview	28	0,03	closed	local	relative	average reference	1.5–6	6.5–8	8.5–12	12.5–30	n/a
Plante 2013	GC	14	22,4 ± 2,8	64	n/a	14	22,1 ± 2,3	64,3	DSM-IV	HDRS	DSM interview	256	2	open	global	absolute	average reference	n/a	n/a	n/a	14.75–15.75	n/a
Putnam 2008	GC	6	32,6 ± 12,1	67	n/a	7	32,8 ± 11,3	71,4	DSM-IV	BDI	DSM interview	128	8	both	local	absolute	average reference	n/a	n/a	8–13	n/a	n/a
Quinn 2014	GC	117	n/a	n/a	n/a	120	n/a	n/a	DSM-IV	HDRS	DSM interview	26	2	closed	local	absolute	averaged A1, A2	n/a	n/a	8–13	n/a	n/a
Roh 2019	both	67	38,39 ± 10,34	87	n/a	60	34,83 ± 9,4	83,3	DSM-IV	HDRS	DSM interview	62	6	both	local	absolute	Cz, CPz	1–4	4–8	8–12	12–30	30–50
Roh 2016	CORR	73	38,71 ± 13,85	55	n/a		n/a	n/a	DSM-IV	HDRS	DSM interview	62	6	both	global	absolute	Cz, CPz	1–4	4–8	8–12	12–30	30–50
Saletu 2010	both	60	51,1 ± 3,13	100	n/a	35	n/a	51,4	ICD-10	HDRS	ICD interview	19	4	closed	local	absolute	n/a	n/a	6–8	n/a	n/a	n/a
Scanlon 2017	CORR	64	43,8 ± 13,3	66	n/a		n/a	n/a	DSM-IV	HDRS	DSM interview	35	0,5	closed	local	absolute and relative	averaged A1, A2	n/a	4–8	8–12	n/a	n/a
Segrave 2011	GC	16	40,75 ± 11,39	100	n/a	18	42,11 ± 13,02	100	DSM-IV	MADS	DSM interview	64	3	both	local	absolute	average reference	n/a	n/a	8–13	n/a	n/a
Soukhtanlou 2019	GC	20	25,1 ± 4,15	50	n/a	20	26 ± 4,63	50	DSM-V	BDI	DSM interview	24	3,5	closed	local	absolute	earlobe electrode	n/a	n/a	8–12	16–30	n/a
Strelets 2006	GC	20	35,95 ± 10,04	70	n/a	28	20,45 ± 3,14	64,3	ICD-10	n/a	ICD interview	16	1,3	closed	local	absolute	earlobe electrode	n/a	n/a	n/a	n/a	30–40
Tas 2014	CORR	25	38,21 ± 12,01	59	4.8 ± 4.34		n/a	n/a	DSM-IV	BDI	DSM interview	19	n/a	closed	local	absolute and relative	averaged A1, A2	n/a	8–12	8–12	n/a	n/a
Wu 2022	both	41	66,41 ± 6,66	81	n/a	44	68,11 ± 7,59	77,3	DSM-IV	HDRS	DSM interview	64	6	both	local	absolute	average reference	1–4	4–8	8–12	12–30	n/a
Xia 2024	GC	48	21,06 ± 2,6	69	n/a	49	21,53 ± 2,18	53	DSM-IV	HDRS	DSM interview	64	n/a	n/a	global	absolute	average reference	1–4	4–8	8–13	13–30	30–70
Zeng 2023	GC	33	68,55 ± 4,92	69	n/a	40	71,2 ± 5,77	62,5	DSM-IV	HDRS	DSM interview	64	5	closed	global	absolute	average reference	n/a	4–8	n/a	n/a	30–66
Zhou 2023	CORR	155	72,9 ± 35,14	73	n/a		n/a	n/a	ICD-10	SRDS	ICD interview	128	5	closed	local	absolute	average reference	1–3	4–6	7–13	14–30	30–100
Zoon 2013	CORR	107	38,95 ± 13,13	58	n/a		n/a	n/a	DSM-IV	HDRS	DSM interview	26	2	closed	local	absolute	earlobe electrode	1.5–3.5	4–7.5	8–13	14.5–30	n/a

GC = Group Comparison (Patients vs. healthy participants, CORR = Correlation, HDRS = Hamilton Depression Rating Scale, BDI = Beck’s Depression Inventory, MDI = Major Depression Inventory (ICD-10), MADS = Montgomery-Asberg Depression Scale, SCL = Symptom Checklist-90 Revised, SRDS = Self-rating depression scales score, POMS = Profile of mood states, n/a = not available.

### 2.3 Record screening, full-text review, and data extraction

Titles and abstracts were independently screened by two authors blinded to each other’s decision. In case of disagreement, conflicts were discussed and resolved. This was performed likewise for full-text review. One author performed the extraction, and another author verified the results. The data extracted comprised general study information, including participant details, EEG recording specifications, and outcome measures.

For cross-sectional group comparisons of EEG features (t-tests and Mann-Whitney-U-tests), the following parameters were extracted for *meta*-analysis: Means and standard deviations (SDs), t-values, U-values, and p-values.

For correlations of disease severity with EEG features (Pearson or Spearman correlations), r-/rho- and p-values were extracted.

If necessary, algebraic recalculation of means, SDs, and effect sizes was implemented following recent recommendations (Covidence, 2021). Data was extracted from figures whenever essential and possible. We contacted study authors to retrieve statistics whenever algebraic recalculation was mathematically impossible. In the case of multiple comparisons reported for one EEG feature (e.g., a study had analyzed several regions of interest for oscillatory theta power), the most significant effect was selected for further analysis. If imprecise p-values for significant findings were reported (for example, “*p* < *0.01*”), we used the closest decimal value for extraction (“*p* = *0.009*”). If p-values for non-significant findings were reported (e.g., “*p* > *0.05*”), we chose not to extract the nearest decimal since a valid approximation of the measured effect could not be guaranteed but included results in the corresponding (“n.s.”) section of the albatross plots. For studies examining alpha asymmetry, correction for the directionality of effects (right-left) was performed in accordance with previous systematic reviews (Luo et al., 2025). Thus, negative values indicate increased left-sided oscillatory alpha activity, and vice versa.

### 2.4 Data synthesis

A multi-step approach was employed for data synthesis, taking into account the number and quality of studies.

First, semiquantitative analyses were performed using modified albatross plots and vote counting, as previously reported, for all comparisons and correlations of interest (Heitmann et al., 2023, Zebhauser et al., 2023). For correlations, albatross plots show p-values for negative correlations with disease severity on the left side of the panel and positive correlations on the right side, respectively. This allowed for the inclusion of studies that only provided imprecise p-values, as reported above.

Second, a *meta*-analysis was performed if k > 4 studies reporting the necessary precise study information were available for the corresponding group comparison or correlation, following recent recommendations on the minimum number of studies needed for random-effect *meta*-analysis (Jackson and Turner, 2017). Meta-analysis was performed using R Version 4.1.2 (R Core Team, 2021) with the *metafor* package (Viechtbauer, 2010). Random-effect models were chosen due to anticipated significant between-study heterogeneity in (i) EEG data acquisition and analysis and (ii) clinical characteristics of study participants. Heterogeneity was evaluated with Cochran’s *Q* (*p* < 0.05 indicating heterogeneity) and *I^2^* (values of 25%, 50%, and 75% representing low, moderate, and high heterogeneity, respectively). Funnel plots and Egger’s tests were used to assess publication bias.

For group comparisons, Hedges’ g was used to compare EEG features between groups due to the small sample sizes of studies. To that end, for studies using parametric statistical tests (t-tests), effect sizes were calculated directly from means and SDs, p-values, and sample sizes. For studies using non-parametric tests (Mann-Whitney U-tests), eta-squared was calculated as an effect size estimate (Fritz et al., 2012) and converted to Hedges’ g using the *esc* package in R as previously reported (Zebhauser et al., 2024). For correlation studies, r- and rho-values were used. Narrative data synthesis was used for the remaining studies.

### 2.5 Risk of bias and quality assessment

The risk of bias (RoB) and study quality were assessed using a modified Newcastle-Ottawa-Scale in terms of “selection of study participants,” “comparability/ confounders,” and “outcome data” (see supplementary material). In the original scale version, stars were awarded for individual domains, whereas the present version used rated items as “high” or “low” RoB for a more straightforward interpretation.

### 2.6 Missing data and full texts

Corresponding authors were contacted up to two times via email to request missing data or inaccessible full texts. Data/full texts were considered unavailable if no reply was received four weeks after the second contact attempt.

## 3 Results

### 3.1 Study selection and characteristics

**Fig. 1:**
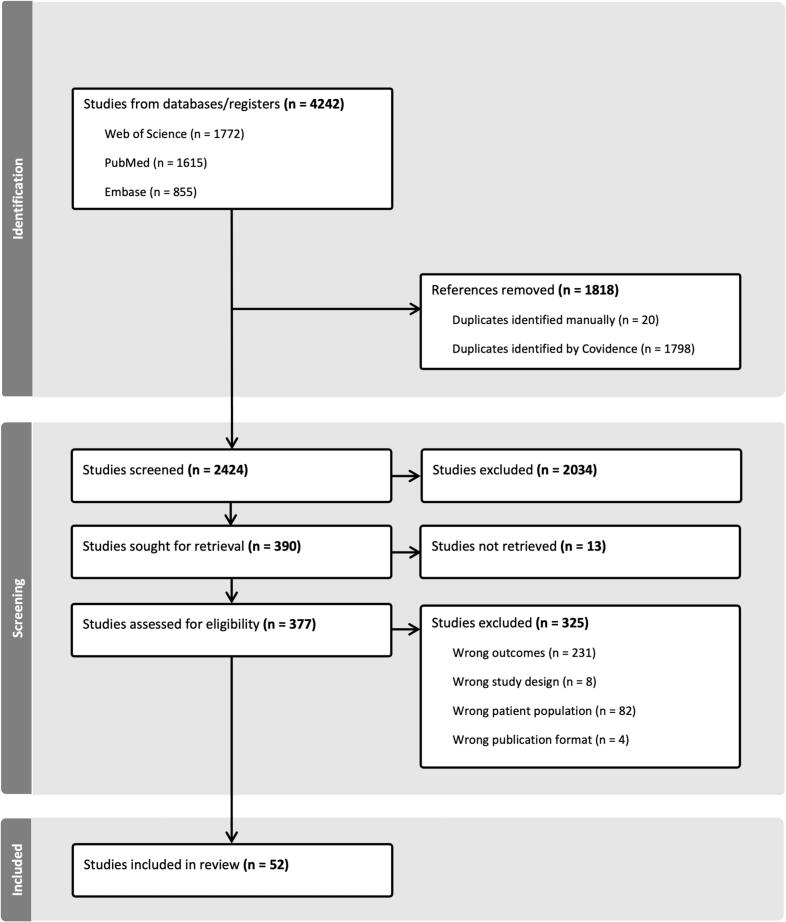
PRISMA-flowchart.

The initial database search yielded 4242 records. Of those, 1818 were identified as duplicates, leaving a total of 2424 records after deduplication. Screening identified 390 studies, of which 52 were finally included (Dharmadhikari et al., Knott et al., 2000, Knott et al., 2001, Pizzagalli et al., 2002, Allen et al., 2004, Morgan et al., 2005, Strelets et al., 2007, Korb et al., 2008, Putnam and McSweeney, 2008, Allen and Cohen, 2010, Farahbod et al., 2010, Kemp et al., 2010, Saletu et al., 2010, Begić et al., 2011, Segrave et al., 2011, Jaworska et al., 2012, Gold et al., 2013, Plante et al., 2013, Cook et al., 2014, Escolano et al., 2014, Quinn et al., 2014, Arns et al., 2015a, Arns et al., 2015b, Cantisani et al., 2015, Tas et al., 2015, Arns et al., 2016, Roh et al., 2016, Scanlon et al., 2016, Mumtaz et al., 2017, Arikan et al., 2019, Kim et al., 2019, Koo-Poeggel et al., 2019, Soukhtanlou et al., 2019, Čukić et al., 2020, Das and Yadav, 2020, Roh et al., 2020, Hill et al., 2021, Lin et al., 2021, Kesebir et al., 2022, Liu et al., 2022a, Liu et al., 2022b, Wu et al., 2022, Huang et al., 2023, Jang et al., 2023, Jiang et al., 2023, Lin et al., 2023, Marcu et al., 2023, Xia et al., 2023, Liu et al., 2024, Zeng et al., 2024). The PRISMA flow diagram of study selection and exclusion reasons at the different levels is shown in Fig. 1. For detailed information, e.g., on the sample characteristics and EEG recording specifications of the studies included, please see Table 2.

### 3.2 Risk of bias and study quality

**Fig. 2:**
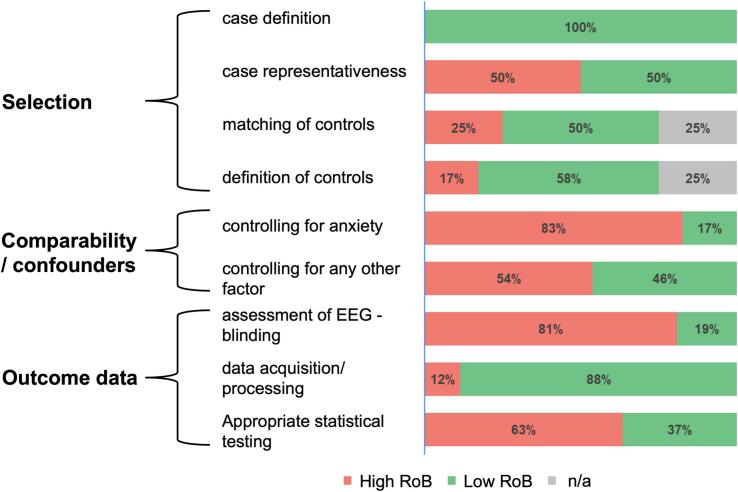
Summary of the Risk of bias (RoB) assessment.

An overview of the results of the risk of bias (RoB) assessment is provided in Fig. 2. The individual studies’ scoring can be found in the supplementary material (Supplementary Table). In the “selection of participants”-domain, the most significant RoB was related to “case representativeness”, as half of the studies did not describe their sampling strategy in detail. Case definition was unproblematic given the strict inclusion criteria for studies regarding diagnostic criteria of patient samples. In the “comparability/confounders”-domain, there was considerable RoB regarding the lack of controlling for anxiety as the most frequent comorbidity of depression and other potential confounding factors such as other comorbidity or medication intake. For the “outcome data”-domain, a high RoB was obtained related to a lack of blinding for the outcome assessment and a partially limited description of the statistical testing applied.

Overall, there was considerable RoB (>50% of studies) in 5/9 domains, with a lack of controlling for relevant comorbidity and non-blinded EEG assessment being the most prominent. For a detailed RoB on the study level, please see Supplementary Table.

## 4 Data synthesis

Two types of data were included in the analyses. Data from cross-sectional studies comparing EEG parameters in patients with depression compared to healthy participants, as well as data from studies reporting the correlation of depression severity with EEG parameters. Data analysis and synthesis followed a two-step approach in the case of four or more studies providing data for the respective EEG parameters. First, as previously reported, semiquantitative data analyses were performed using modified albatross and vote counting (Heitmann et al., 2023, Zebhauser et al., 2023). Second, a *meta*-analysis was performed if four or more studies provided sufficient data (Zebhauser et al., 2024). For less frequently reported EEG parameters, narrative synthesis was performed.

### 4.1 Group comparisons (depression vs. Healthy participants)

A total of 33 studies performed cross-sectional group comparisons of one or more EEG parameters in patients with depression compared to healthy participants. Of those, 26 studies reported a comparison of band-specific EEG power (Dharmadhikari et al., Knott et al., 2001, Morgan et al., 2005, Strelets et al., 2007, Korb et al., 2008, Begić et al., 2011, Jaworska et al., 2012, Zeng et al., 2012, Plante et al., 2013, Cook et al., 2014, Arns et al., 2015a, Arns et al., 2015b, Mumtaz et al., 2017, Arikan et al., 2019, Soukhtanlou et al., 2019, Čukić et al., 2020, Das and Yadav, 2020, Hill et al., 2021, Lin et al., 2021, Liu et al., 2022a, Liu et al., 2022b, Wu et al., 2022, Huang et al., 2023, Jang et al., 2023;, Jiang et al., 2023, Lin et al., 2023, Xia et al., 2023). Comparison of alpha asymmetry (Luo et al., 2025) (AA) was reported in 14 studies (Dharmadhikari et al., Knott et al., 2001, Allen and Cohen, 2010, Kemp et al., 2010, Segrave et al., 2011, Jaworska et al., 2012, Cantisani et al., 2015, Arns et al., 2016, Mumtaz et al., 2017, Koo-Poeggel et al., 2019, Roh et al., 2020, Lin et al., 2021, Wu et al., 2022, Liu et al., 2024). Additionally, Peak-Alpha Frequency (PAF) (Arns et al., 2015a, Arns et al., 2015b) and cordance (Cook et al., 2014) were compared between patients with depression and healthy participants in one study, respectively.

### 4.2 Semiquantitative analyses

**Fig. 3:**
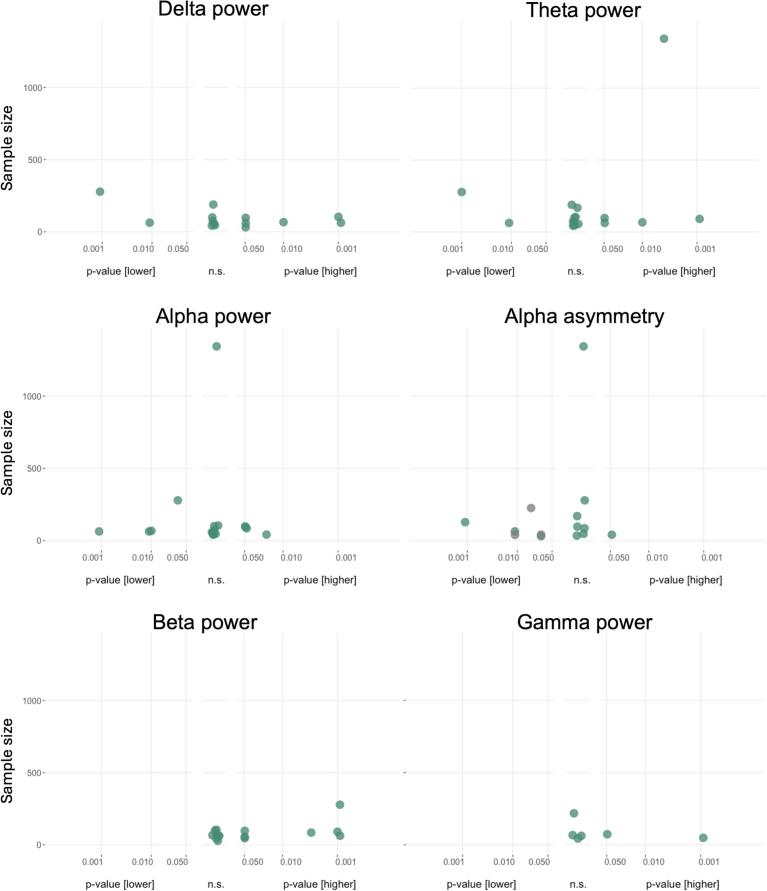
**Albatross plots of group comparisons in band-specific power and alpha asymmetry.** Power differences for group comparisons between patients and healthy participants. P values on the x-axis are displayed on a logarithmic scale (log10). Higher values in patients compared to healthy participants are depicted on the right-hand side, non-significant differences in the middle and lower values on the left-hand side of each panel. The total sample size for single studies is depicted on the y-axis. n.s., not significant. Due to the adaptation of directionality in alpha asymmetry (right-left), lower values indicate a predominance of left-sided frontal alpha activity.

Fig. 3 shows the results of group comparisons for band-specific power and alpha asymmetry.

*Power* in the *delta band* was compared by 13 studies. Six studies reported higher (Morgan et al., 2005, Begić et al., 2011, Čukić et al., 2020, Das and Yadav, 2020, Huang et al., 2023, Xia et al., 2023), five reported no difference (Knott et al., 2001, Korb et al., 2008, Liu et al., 2022a, Liu et al., 2022b, Jiang et al., 2023), and two had lower (Mumtaz et al., 2017, Lin et al., 2021) values for patients than healthy participants. *Theta band* power was compared in 16 studies. Five found higher values (Begić et al., 2011, Arns et al., 2015a, Arns et al., 2015b, Huang et al., 2023, Lin et al., 2023, Xia et al., 2023), nine no differences (Knott et al., 2001, Morgan et al., 2005, Korb et al., 2008, Cook et al., 2014, Das and Yadav, 2020, Hill et al., 2021, Liu et al., 2022a, Liu et al., 2022b, Jiang et al., 2023), and two studies found lower patient values (Mumtaz et al., 2017, Lin et al., 2021). Seventeen studies reported comparisons of *alpha power.* Four reported higher values (Dharmadhikari et al., Jaworska et al., 2012, Wu et al., 2022, Xia et al., 2023), nine reported no difference (Knott et al., 2001, Morgan et al., 2005, Korb et al., 2008, Arns et al., 2015a, Arns et al., 2015b, Mumtaz et al., 2017, Das and Yadav, 2020, Hill et al., 2021, Liu et al., 2022a, Liu et al., 2022b), and four reported lower values (Begić et al., 2011, Mumtaz et al., 2017, Lin et al., 2021, Huang et al., 2023) in patients. *Beta power* was assessed in 15 studies. Seven studies found higher values (Knott et al., 2001, Begić et al., 2011, Lin et al., 2021, Liu et al., 2022a, Wu et al., 2022, Lin et al., 2023, Xia et al., 2023), eight did not find a difference (Morgan et al., 2005, Korb et al., 2008, Plante et al., 2013, Mumtaz et al., 2017, Das and Yadav, 2020, Hill et al., 2021, Liu et al., 2022b, Huang et al., 2023), and none found lower values in patients compared to healthy participants. Six studies reported comparisons in *gamma power,* with two studies showing higher values (Strelets et al., 2007, Zeng et al., 2024), four studies showing no difference (Arikan et al., 2019, Hill et al., 2021, Huang et al., 2023, Jang et al., 2023), and none showing lower values.

*Alpha asymmetry* was compared between patients and healthy controls in 14 studies, all of which reported results from frontal brain regions/electrodes. One study found higher values, i.e. higher right-sided alpha activity (Koo-Poeggel et al., 2019), seven found no difference (Knott et al., 2001, Segrave et al., 2011, Jaworska et al., 2012, Arns et al., 2016, Lin et al., 2021, Wu et al., 2022, Liu et al., 2024), and six found lower values, i.e. higher left-sided alpha activity, in patients than healthy participants (Dharmadhikari et al., Allen and Cohen, 2010, Kemp et al., 2010, Cantisani et al., 2015, Mumtaz et al., 2017, Roh et al., 2020).

Thus, semiquantitative analyses show a trend towards increased low (delta and theta) and high (beta and gamma) frequency band power in patients. Moreover, for alpha asymmetry, a trend toward a relative increase in left-sided oscillatory activity is observed in patients compared to healthy participants.

### 4.3 Meta-analysis

**Fig. 4:**
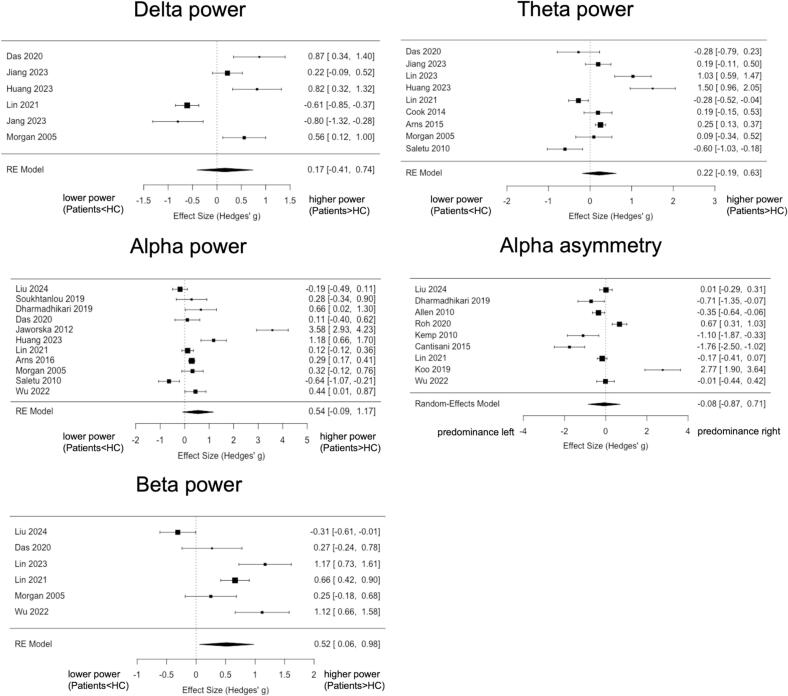
**Forest plots of *meta*-analysis for comparisons of band-specific power and alpha asymmetry.** For band-specific power, positive values favour higher power in patients, and negative values favour lower power in patients, compared to healthy controls/participants (HC) respectively. Due to the adaptation of directionality in alpha asymmetry (right-left), negative values indicate a predominance of left-sided frontal alpha activity. RE = random effects. HC = healthy controls/participants.

If four or more studies provided sufficient information, a *meta*-analysis was performed. Fig. 4 shows the results for band-specific power and alpha asymmetry. Meta-analysis was not possible for *gamma* power since sufficient information was not available.

*Band-specific power* comparison in the *delta* band yielded non-significant results with a high degree of heterogeneity among studies (k = 6 studies, *Hedges’ g* = *0.17*, *95% CI −0.41*–*0.74*; heterogeneity: *I^2^* = *92.3%*, *p [Q]* < *0.001*). A similar picture was obtained for *theta* (k = 9 studies, *Hedges’ g* = *0.22*, *95% CI −0.19*–*0.63*; heterogeneity: *I^2^* = *93.7%*, *p [Q]* < *0.001*) and alpha power (k = 12 studies, *Hedges’ g* = *0.50*, *95% CI −0.07*–*1.08*; heterogeneity: *I^2^* = *97.3%*, *p [Q]* < *0.001*). For *beta power,* a significant group difference was obtained, with higher values in patients compared to healthy participants (k = 6 studies, *Hedges’ g* = *0.52*, *95% CI −0.06*–*0.98*; heterogeneity: *I^2^* = *88.9%*, *p [Q]* < *0.001*) but considerable heterogeneity.

*Alpha asymmetry* did not differ between groups (k = 9, *Hedges’ g* = -*0.08*, *95% CI −0.87*–*0.71*; heterogeneity: *I^2^* = *81.4%*, *p [Q]* < *0.001*), and heterogeneity was high.

Funnel plots suggested some asymmetry, especially for alpha power, but Egger’s test did not show evidence of publication bias (p > 0.5) in all group comparisons (Supplementary Fig. S1).

In summary, a *meta*-analysis confirmed an increase in beta power in patients with depression compared to healthy participants.

### 4.4 Narrative synthesis

Regarding group comparison of other EEG parameters, one study reported that PAF did not differ between the two groups (Arns et al., 2015a, Arns et al., 2015b). Moreover, one study found that *cordance* was higher in patients than in healthy participants (Cook, Hunter et al., 2014), especially in the theta band.

### 4.5 Correlations with disease severity

#### 4.5.1 Semiquantitative analyses

**Fig. 5:**
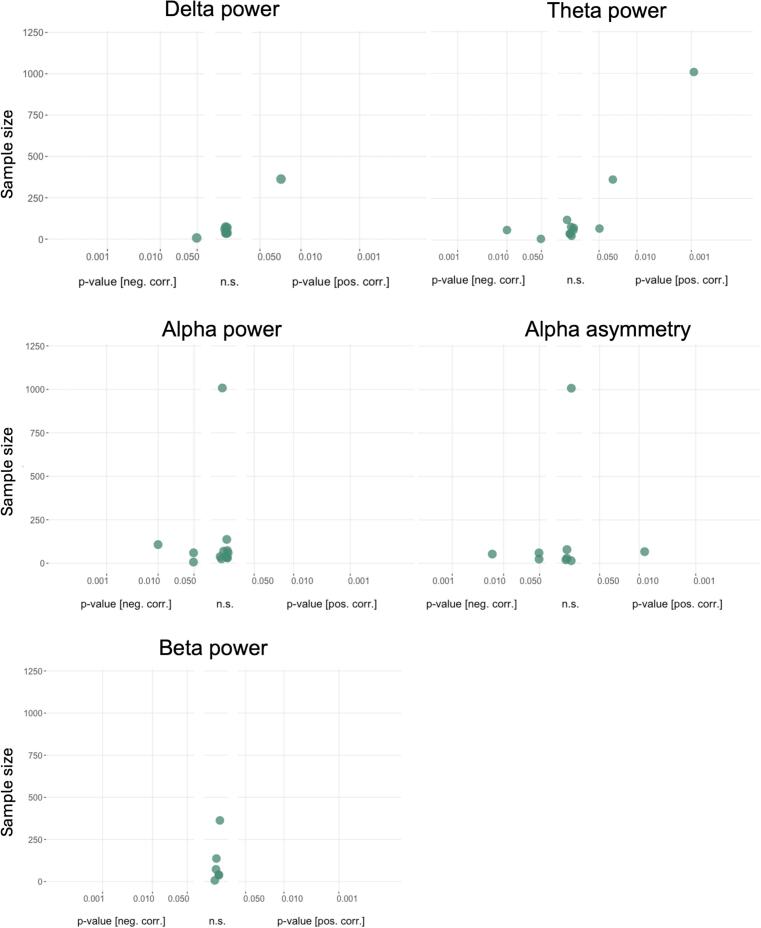
**Albatross plots for band-specific power and alpha asymmetry correlations with disease severity.** Plots show correlations of corresponding parameters with disease severity. P values of correlations are displayed on the x-axis on a logarithmic scale (log10). Positive correlations are depicted on the right-hand side, non-significant differences in the middle, and negative correlations on the left-hand side of each panel. The sample size for single studies is depicted on the y-axis. n.s., not significant.

Fig. 5 summarizes the results of the correlation analysis of band-specific power and alpha asymmetry with disease severity. The number of studies available for *gamma* power was insufficient for semiquantitative analysis.

Correlations between *band-specific power* in the *delta* range and depression severity were assessed in eight studies. One study showed a positive (Kesebir et al., 2022), one study a negative (Farahbod et al., 2010), and six studies no significant correlation (Knott et al., 2001, Korb et al., 2008, Escolano et al., 2014, Roh et al., 2016, Kim et al., 2019, Huang et al., 2023). Twelve studies performed correlations for theta power. Three reported positive (Knott et al., 2001, Arns et al., 2015a, Arns et al., 2015b, Kesebir et al., 2022), two negative (Farahbod et al., 2010, Saletu et al., 2010), and seven no significant correlation (Korb et al., 2008, Gold et al., 2013, Cook et al., 2014, Escolano et al., 2014, Tas et al., 2015, Roh et al., 2016, Kim et al., 2019). A total of 13 studies tested for correlations between power in the *alpha* range and depression severity. No study found a positive correlation; three reported negative correlations (Farahbod et al., 2010, Saletu et al., 2010, Zoon et al., 2013), and 10 reported non-significant correlations (Knott et al., 2001, Korb et al., 2008, Escolano et al., 2014, Tas et al., 2015, Arns et al., 2016, Roh et al., 2016, Kim et al., 2019, Das and Yadav, 2020, Lin et al., 2021, Huang et al., 2023). Six studies performed correlations with *beta power*, and all reported non-significant results (Farahbod et al., 2010, Roh et al., 2016, Kim et al., 2019, Lin et al., 2021, Kesebir et al., 2022, Wu et al., 2022).

Correlations between *alpha asymmetry* and depression severity were assessed in nine studies, all of which reported results from frontal brain regions/electrodes. Of those, one study reported a positive (Roh et al., 2020), three a negative (Saletu et al., 2010, Jaworska et al., 2012, Marcu et al., 2023), and five a non-significant relationship (Allen et al., 2004, Kemp et al., 2010, Gold et al., 2013, Cantisani et al., 2015, Arns et al., 2016).

In summary, semiquantitative analyses do not point towards a consistent relationship between band-specific power and alpha asymmetry with depression severity.

### 4.6 Meta-analysis

**Fig. 6:**
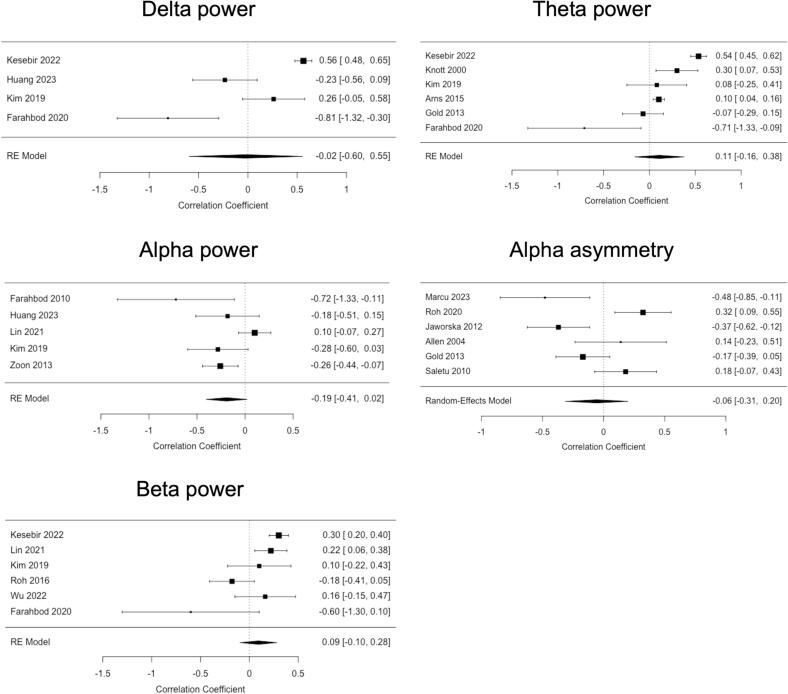
**Forest plots of *meta*-analysis for correlations of band-specific power and alpha asymmetry with disease severity.** RE = random effects.

If four or more studies provided sufficient information, a *meta*-analysis was performed. Fig. 6 shows the results for band-specific power and alpha asymmetry. Meta-analysis was not possible for *gamma* power since sufficient information was not available.

Correlations between depression severity and *band-specific power* in the *delta* (k = 4 studies, *r* = -*0.02*, *95% CI −0.60*–*0.55*; heterogeneity: *I^2^* = *94.3%*, *p [Q]* < *0.001*) and *theta* band (k = 6 studies, *r* = *0.11*, *95% CI −0.16*–*0.38*; heterogeneity: *I^2^* = *94.8%*, *p [Q]* < *0.001*) were non-significant and showed very high heterogeneity. Correlations with *alpha* power were also non-significant and showed moderate heterogeneity (k = 5 studies, *r* = *-0.19*, *95% CI −0.41*–*0.02*; heterogeneity: *I^2^* = *67.5%*, *p [Q]* < *0.01*). For *beta power,* a non-significant correlation was obtained, showing moderate heterogeneity (k = 6, *r* = -*0.09*, *95% CI −0.10*–*0.28*; heterogeneity: *I^2^* = *75.4%*, *p [Q]* < *0.01*).

*Alpha asymmetry* was not correlated with depression severity (k = 6, *r* = -*0.06*, *95% CI −0.31*–*0.20*; heterogeneity:*I^2^* = *81,4%*, *p [Q]* < *0.001*), and heterogeneity was high.

Funnel plots suggested some asymmetry, especially for delta and beta power. However, Egger’s test did not show evidence of publication bias (p > 0.5) in the reported correlations (Supplementary Fig. S2).

Taken together, no consistent relationship of band-specific power and alpha asymmetry with depression severity was found in the semiquantitative and *meta*-analyses.

### 4.7 Narrative synthesis

*PAF* was related to depression severity in two studies. One study reported a significant positive correlation (Zhou et al., 2023) and another no relationship (Arns et al., 2015a, Arns et al., 2015b).

Correlations of *cordance* with depression severity were performed in two studies. One study reported significant correlations of *cordance* in the alpha band with depression severity but with opposing directionality for different brain regions and non-significant correlations in the theta band (Scanlon et al., 2016). The other study reported no significant correlation between *cordance* and depression severity (Cook et al., 2014).

## 5 Discussion

The present study found *meta*-analytic evidence for an increase in the power of beta-oscillations and semiquantitative evidence for an increase in slow frequency oscillations in the delta and theta range, as well as alpha asymmetry with relatively increased left-sided frontal oscillatory activity in patients with depression compared to healthy participants.

### 5.1 Oscillatory brain activity in depression

The semiquantitative and *meta*-analytic results presented here in principle align with findings from previous narrative reviews reporting an increase in delta, theta, and beta oscillations (Newson and Thiagarajan, 2019) as well as a potential role for slow (mainly theta) and high (primarily gamma) frequency oscillations and a recent *meta*-analysis pointing towards left frontal alpha asymmetry as potential diagnostic biomarkers in depression (de Aguiar Neto and Rosa, 2019, Tsai et al., 2023, Luo et al., 2025).

*Beta oscillations* have been implicated in the top-down control of cortico-limbic circuits in depression (Hoy et al., 2023, Amemori et al., 2024, Xiao et al., 2024). They appear to be specifically involved in reward learning and reward biases in patients with depression and have been discussed as a potential biomarker of anhedonia (Xiao et al., 2024). Furthermore, experimental evidence shows that *beta oscillations* determine effort-related aspects and *theta oscillations* reward-related aspects of reward learning in depression. Additionally, increased *theta oscillations* were associated with negative experiences in patients with depression (Riddle et al., 2020) and differentiated patients with MDD from those with anxiety disorder (Zhang et al., 2022). The role of theta oscillations in anxiety, as the most prominent comorbidity of depression, remains to be elucidated and warrants further study (Newson and Thiagarajan, 2019). *Theta oscillations* have also been previously discussed as potential monitoring biomarkers in depression, e.g., predicting therapeutic response for non-invasive brain stimulation (Bailey et al., 2018), and thus as a treatment target (Tsai et al., 2023). However, the present study does not find a relationship between brain oscillations at any frequency and disease severity, suggesting limited potential as a monitoring biomarker. This does not mean that such a relationship does not exist, but that the current systematic review and *meta*-analysis of existing evidence cannot detect it.

*Alpha asymmetry* in depression has been conceptually related to an imbalance in appetitive and aversive motivational processing, which may correspond to negative and positive affect (van der Vinne et al., 2017). A widely described predominance of left-sided frontal alpha activity was found to be correlated with sensitivity of the behavioral activation system (i.e., a deficiency in motivation) and hypothesized to be associated with anhedonia in previous literature (van der Vinne et al., 2017). Our inconclusive findings regarding left alpha asymmetry reflect the prior literature, including recent systematic reviews and *meta*-analyses, with some showing such an effect (Luo et al., 2025) and others not (van der Vinne et al., 2017). Both previous *meta*-analyses critically discuss the specificity and the role of methodological heterogeneity in left frontal alpha asymmetry, thereby questioning its use as a (single) diagnostic biomarker for depression.

Thus, our data point towards a potential role for beta oscillations in depression. Evidence for the previously reported alterations in slow frequency oscillations and alpha asymmetry remains inconclusive in the present study.

### 5.2 Specificity and potential transdiagnostic implications

Similar to the present and previous observations discussed in patients with depression, combinations of increases in slow (especially theta) and high (especially beta) frequencies have been described in patients with chronic pain and pathological fatigue, thereby questioning the specificity of the findings (Heitmann et al., 2023, Zebhauser et al., 2023, Zebhauser et al., 2024). Even though a potential lack of specificity limits the applicability as a biomarker for a single disease entity, a transdiagnostic view, e.g., at the circuit level, might even open new avenues for future treatments in neuropsychiatric disorders (Scangos et al., 2023). Depression, fatigue, and pain are highly comorbid and overlap in their anhedonic valence, which has fostered a discussion of reward deficiency as a potential common underlying pathological mechanism (Heitmann et al., 2020). A common electrophysiological model explaining this comorbidity is thalamocortical dysrhythmia. This model proposes that abnormal thalamocortical theta oscillations cause alterations in higher frequency bands in the beta and gamma range, resulting in different neuropsychiatric symptoms, including pain and depression (Llinas et al., 1999, Sarnthein et al., 2005, Schulman et al., 2011, De Ridder and Vanneste, 2024). Recently, this model was also applied to neuropsychiatric disorders associated with reward deficiency and dopaminergic dysfunction, highlighting a role for theta and beta oscillations in the anterior cingulate cortex (ACC) and ventromedial prefrontal cortex (vmPFC) in these conditions (De Ridder and Vanneste, 2024). This further supports the notion that aberrant theta and beta oscillations might reflect a common transdiagnostic pathomechanism related to altered reward processing and dopaminergic dysfunction across various disorders.

### 5.3 Risk of bias and limitations

Different factors limit the interpretation and generalizability of the present results. They are mainly related to the included studies and the analyses applied.

First, there was a considerable RoB, primarily due to a lack of control for relevant comorbidity, especially anxiety, potential medication effects on EEG activity (Aiyer et al., 2016), and non-blinded EEG assessment. Second, the sample sizes of the studies were relatively small (median n = 68). The effects reported were observed in group comparisons, where sample size calculation indicates that for detecting medium effect sizes with a power of 80% (two-tailed *t*-test, *alpha* = 0.05, *1-beta* = 0.8), a total sample size of n = 128 would be appropriate (Faul et al., 2007). Thus, most studies included were underpowered, increasing the risk of false-negative and false-positive findings (Button et al., 2013). Third, there was considerable methodological heterogeneity, especially regarding outcome parameters. However, due to the relatively small number of studies, results had to be pooled irrespective of the methods applied, e.g., local and global power results, data from eyes open and eyes closed EEG measurements, and using different recording systems and electrode placements. This can obscure specific effects but render the results obtained despite this heterogeneity more robust. However, even though the majority of studies included reported global power results, deciding to include only the strongest effect when multiple results across different brain regions are reported introduces a potential selection bias toward false positives. Fourth, the number of included studies varied substantially across EEG measures. Only half of the studies could be included in *meta*-analyses for some EEG parameters, potentially introducing bias. For example, semiquantitative analyses pointed toward increased delta and theta oscillations, but *meta*-analyses did not yield significant results. However, for delta oscillations, only 3/6 studies, and for theta, only 3/5 studies reporting higher power in patients, could be included in the *meta*-analyses due to a lack of detailed information required for these analyses. Still, for both frequency bands, 2/2 studies reporting lower power were included. This was vice versa for alpha power. Here, only 1/4 studies from the semiquantitative analyses reporting lower, but 4/4 studies reporting higher power in patients could be included in the *meta*-analysis, which then suggested a trend towards higher alpha power. Fifth, the lack of correlation between beta power and disease severity limits its potential as a biomarker for diagnostic, but not monitoring, purposes. Sixth, the insights from this study focus on EEG power measures and do not cover other important and increasingly examined EEG parameters in depression, e.g., connectivity measures, 1/f non-oscillatory brain activity, which potentially also influences oscillatory brain activity, and microstate analyses. However, as stated in the introduction, the focus on frequently analyzed EEG parameters was chosen to maximize the number of studies included, thereby allowing for *meta*-analysis.

### 5.4 Outlook and recommendations

Considering the gaps in current methods and findings outlined above, this systematic review might help to guide future studies evaluating the potential of EEG-based biomarkers in depression. The high risk of bias arising from methodological heterogeneity warrants further efforts to standardize clinical assessments, e.g., to include relevant comorbidities, as well as the recording and analysis of EEG data. Biomarker discovery in neuropsychiatric disorders critically depends on reproducible, transparent methods applied to large-scale datasets (Gil Avila et al., 2023). This should include performance and reporting of studies in line with consensus statements (Pernet et al., 2020a), standardized structuring of data (e.g. EEG-BIDS) (Pernet et al., 2019), and the use of pipelines allowing for automatic preprocessing and analysis of EEG data (Pedroni et al., 2019, Pernet et al., 2020b, Gil Avila et al., 2023). This also facilitates the comparison or even pooling of results from smaller individual datasets (Bott et al., 2025).

## 6 Conclusions

The present systematic review and *meta*-analysis provide an overview of EEG activity-based measures in depression, yielding evidence for increased beta power and inconclusive evidence for increased slow-frequency power and left frontal alpha asymmetry in patients with depression. This provides insights into the pathophysiology of depression. However, the high RoB of studies highlights the need for well-powered and standardized studies to further evaluate the potential of these parameters as biomarkers or even future treatment targets in depression.

## CRediT authorship contribution statement

**Henrik Heitmann:** Writing – original draft, Visualization, Validation, Project administration, Methodology, Investigation, Formal analysis, Data curation, Conceptualization. **Jean-François Siani:** Writing – original draft, Project administration, Formal analysis, Data curation, Conceptualization. **Paul Theo Zebhauser:** Writing – review & editing, Validation, Methodology, Investigation, Conceptualization. **Peter Henningsen:** Writing – review & editing, Supervision. **Stefan Leucht:** Writing – review & editing, Validation, Supervision, Methodology. **Josef Priller:** Writing – review & editing, Supervision, Conceptualization. **Markus Ploner:** Writing – review & editing, Validation, Supervision, Resources, Methodology, Investigation, Funding acquisition, Conceptualization.

## Funding

The present work was funded by the TUM Innovation Network for Neurotechnology in Mental Health (NEUROTECH).

## Declaration of competing interest

The authors declare that they have no known competing financial interests or personal relationships that could have appeared to influence the work reported in this paper.

## Data Availability

Data will be made available on request.

## Appendix A Supplementary data

Supplementary Data 1 Supplementary Data containing detailed information on search strategy and risk of bias assessment as well as Funnel plots.
